# Impact of Transcranial Direct Current Stimulation and Cognitive Training on Frontal Lobe Neurotransmitter Concentrations

**DOI:** 10.3389/fnagi.2021.761348

**Published:** 2021-10-21

**Authors:** Stacey Alvarez-Alvarado, Emanuel M. Boutzoukas, Jessica N. Kraft, Andrew O’Shea, Aprinda Indahlastari, Alejandro Albizu, Nicole R. Nissim, Nicole D. Evangelista, Ronald Cohen, Eric C. Porges, Adam J. Woods

**Affiliations:** ^1^Center for Cognitive Aging and Memory, McKnight Brain Institute, University of Florida, Gainesville, FL, United States; ^2^Department of Clinical and Health Psychology, University of Florida, Gainesville, FL, United States; ^3^Department of Neuroscience, University of Florida, Gainesville, FL, United States

**Keywords:** transcranial direct current stimulation (tDCS), magnetic resonance spectroscopy (^1^H-MRS), cognitive training, glutamate, gamma-aminobutyric acid (GABA)

## Abstract

**Objective:** This study examines the impact of transcranial direct current stimulation (tDCS) combined with cognitive training on neurotransmitter concentrations in the prefrontal cortex.

**Materials and Methods:** Twenty-three older adults were randomized to either active-tDCS or sham-tDCS in combination with cognitive training for 2 weeks. Active-tDCS was delivered over F3 (cathode) and F4 (anode) electrode placements for 20 min at 2 mA intensity. For each training session, 40-min of computerized cognitive training were applied with active or sham stimulation delivered during the first 20-min. Glutamine/glutamate (Glx) and gamma-aminobutyric acid (GABA) concentrations *via* proton magnetic resonance spectroscopy were evaluated at baseline and at the end of 2-week intervention.

**Results:** Glx concentrations increased from pre- to post-intervention (*p* = 0.010) in the active versus sham group after controlling for age, number of intervention days, MoCA scores, and baseline Glx concentration. No difference in GABA concentration was detected between active and sham groups (*p* = 0.650) after 2-week intervention.

**Conclusion:** Results provide preliminary evidence suggesting that combining cognitive training and tDCS over the prefrontal cortex elicits sustained increase in excitatory neurotransmitter concentrations. Findings support the combination of tDCS and cognitive training as a potential method for altering neurotransmitter concentrations in the frontal cortices, which may have implications for neuroplasticity in the aging brain.

## Introduction

Transcranial direct current stimulation (tDCS) is a non-invasive brain stimulation technique that applies a weak and constant electric current *via* electrodes placed on the scalp over target brain areas (Nitsche and Paulus, 2000; Woods et al., 2016). Although the precise physiological mechanisms underlying tDCS are still unclear, the primary mechanism of action derives from alteration of the resting membrane potential, leading to changes in spontaneous neural firing rate (Nitsche and Paulus, 2000, 2001). These polarity-specific alterations of cortical excitability are predominately demonstrated in motor regions of the brain (e.g., M1) by inducing greater transcranial magnetic stimulation (TMS) produced motor-evoked potential magnitudes of up to 40% (Nitsche and Paulus, 2000). Following current cessation, sustained excitability (e.g., after-effects) of tDCS are demonstrated for 30–90 min (Nitsche and Paulus, 2001; Nitsche et al., 2003; Kidgell et al., 2013; Jamil et al., 2017) and persist up to 24 h when stimulation is applied multiple times during one day (Bastani and Jaberzadeh, 2014). Moreover, extended cortical excitability is evidenced by applying a second consecutive tDCS session during the after-effect window on the motor cortex of healthy humans (Monte-Silva et al., 2013). In part, after-effects are attributed to physiological changes in synaptic plasticity, where excitatory and inhibitory neurotransmitters mediate long-term potentiation. Key markers, particularly gamma-aminobutyric acid (GABA) and glutamate signaling (Trepel, 1998; Froc and Racine, 2004), facilitate the neuroplastic response of tissue and modify the immediate relative contribution to neural events (Krause et al., 2013; Dwyer et al., 2019). These cortical neurotransmitter concentrations can be assessed non-invasively within a defined region of interest in the brain using proton (^1^H) magnetic resonance spectroscopy (MRS) (Stagg et al., 2011; Puts and Edden, 2012), providing insight into the neural bases of cognition (Kanai and Rees, 2011).

The relative concentrations of excitatory/inhibitory neurotransmitters may play a key role in further understanding the tDCS-related neuroplastic effects. GABA is the major inhibitory neurotransmitter in the human brain (McCormick, 1989), essential for synaptic communication and linked to various cognitive functions, including working memory capacity in the prefrontal cortex (Yoon et al., 2016). Brain aging is associated with GABAergic neuroplasticity dysfunction, which relates to global GABA level reduction (Richardson et al., 2013). Meanwhile, the excitatory neurotransmitter, glutamate, acts in most of the excitatory synapses in the central nervous system, while synthesized from glutamine through the action of glutaminase (Fonnum, 1993). Glutamate is often reported as Glx in MRS, representing the combination of glutamate and glutamine as their spectra highly overlap due to similar molecular structures (Harris et al., 2017). Together, GABA and glutamate contribute synergistically to cognitive performance (Jocham et al., 2012), primarily during the initiation of new learning processes by disrupting the excitatory/inhibitory balance. This initial shift toward increased excitatory contributions allows activity-dependent refinements of cortical circuits (Carcea and Froemke, 2013). Particularly, glutamate receptors [i.e., N-Methyl-D-Aspartate-receptor (NMDA)] are best known for mediating glutamate’s role in learning and memory through plasticity of channel properties such as enhancement of glutamate neurotransmission and gene expression (Barco et al., 2006). As learning becomes more efficient over time, GABA ^1^H-MRS concentrations have been shown to increase in task-relevant brain regions (Sampaio-Baptista et al., 2015). Less is known of the tDCS training-induced responses in excitatory/inhibitory neurotransmitter concentrations, particularly relevant for prompting long-term neurocortical adaptations in the aging brain. Since tDCS augments training-induced cognitive gains, investigating these neural mechanisms may provide insight into effective interventions geared toward neuroplasticity and cognitive preservation.

A limited number of studies have investigated the effects of tDCS using ^1^H-MRS. Previous ^1^H-MRS studies primarily investigated single-session tDCS over the motor cortex and found increases in sensorimotor glutamate (Stagg et al., 2011), decreases in GABA concentrations (Stagg et al., 2009, 2011; Kim et al., 2014; Bachtiar et al., 2015; Patel et al., 2019), and no change in either (Tremblay et al., 2016). These transcient increases in excitatory neurotransmitter with concurrent reductions in inhibitory neurotransmitter concentration, following single-session stimulation, are consistent with the neuromodulatory properties of tDCS. Nonetheless, these effects are contingent on the stimulation parameters, targeted cortical area (Dwyer et al., 2019), and baseline properties of the targeted cortical region (Filmer et al., 2019; Indahlastari et al., 2020). The ability to elicit similar facilitatory effects within other cortical areas is, however, less clear. Barron et al. (2016) applied tDCS to the occipital-temporal cortices (anode: occipital-temporal, cathode: supraorbital ridge) and found dormant associative memories can be re-expressed by reduced cortical GABA and increased glutamate concentrations in young adults. Meanwhile, GABA and Glx concentrations were unchanged when targeting the posterior superior temporal gyrus in young adults (Dwyer et al., 2019). Recently, Mezger et al. (2021) investigated the effects of bifrontal stimulation on GABA and Glx concentrations in young adults and found no change, suggesting that a single-session of tDCS may be less effective when targeting non-motor cortex in young, healthy adults. Correspondingly, with recent applications of tDCS to the prefrontal cortex to enhance cognitive training/learning outcomes (Martin et al., 2013; Nissim et al., 2019), there is still need to demonstrate the precise nature and impact of tDCS in the frontal cortex, a brain region highly prone to age-related cognitive decline. Although inducing long-term excitability is valuable in neuroplasticity modulation, the lasting effects of tDCS over multiple sessions remain unknown (Nitsche and Paulus, 2001; Jamil et al., 2017). Further, the long-term maintenance of Glx and GABA effects following multiple sessions of tDCS in older adults has yet to be addressed.

Due to its effects on excitability and synaptic plasticity, prior studies have investigated tDCS as a potential adjunctive tool aimed at facilitating cognitive training effects in older adults (Gill et al., 2015; Jones et al., 2015; Stephens and Berryhill, 2016; Nissim et al., 2019). Significant improvements in behavior have been observed when tDCS is paired with cognitive training in comparison to cognitive training alone, supporting a paired intervention approach (Jones et al., 2015; Stephens and Berryhill, 2016). Further, applying tDCS over relevant brain regions during tasks/activities with higher cognitive demands results in greater potential of improving cognitive outcomes (Woods et al., 2016; Antonenko et al., 2018; Nissim et al., 2019). Combining working memory training with tDCS has shown to extend and increase training gains (Jones et al., 2015; Stephens and Berryhill, 2016). For instance, Stephens and Berryhill (2016) found that older adults receiving active tDCS versus sham during working memory training resulted in greater benefits on untrained assessment tasks post-training. Pairing tDCS and cognitive training has been associated with improved skill acquisition on the cognitive training task when compared to tDCS applied before the training task (Martin et al., 2014). In a recent study, we demonstrated that pairing tDCS (bilaterally to the frontal lobes; F3/F4) with cognitive training for 2 weeks enhanced working memory performance in older adults (Nissim et al., 2019). However, the underlying neural mechanisms linking these improvements in cognitive performance are yet to be elucidated. Because the combination of brain stimulation and multi-session cognitive training may counteract and delay the onset of age-related impairments (Perceval et al., 2016; Indahlastari et al., 2021), understanding the modulatory role of inhibitory and excitatory neurotransmitter concentrations in a multi-day setting is of clinical and scientific relevance.

To be able to draw further conclusion about the efficiency of these paired interventions, controlled randomized clinical trials in cognitively intact older adults are required. In light of this gap in the literature, the goal of the present study was to examine the impact of a 2-week intervention of active-tDCS and sham-tDCS combined with cognitive training on neurotransmitter concentrations in the prefrontal cortex using ^1^H-MRS. We hypothesized increased Glx concentrations coupled with decreased GABA concentrations pre- to post-intervention in the active-tDCS group when compared to the sham-tDCS group.

## Materials and Methods

### Participants

Twenty-eight healthy older adults completed the parent study. This phase II clinical pilot study employed a randomized, triple-blinded (assessor, interventionist, participant) between subjects design, permitting examination of combined effects of tDCS with cognitive training on neurotransmitter concentrations in healthy older adults. Of these, 23 older adults (*n* = 12 active; *n* = 11 sham) completed all MRS/magnetic resonance imaging (MRI) visits and were included in the current study. A prior publication reported functional connectivity from functional MRI (fMRI) and behavioral effects from the parent trial (Nissim et al., 2019). The parent trial was preregistered in clinicaltrials.gov under NCT02137122.

Participants between the ages of 65–88 years with no evidence of cognitive impairment, as defined by the National Alzheimer’s Coordinating Center Uniform Data Set performance below 1.5 standard deviations on age, sex, or education normative data in at least one cognitive domain (Woods et al., 2018), were screened for eligibility. Exclusion criteria included pre-existing neurodegenerative or psychiatric brain disorders (i.e., dementia, Alzheimer’s, schizophrenia), MRI contraindications (i.e., metal or medical devices inside body not approved to be scanned at 3T), ineligibility for tDCS scalp application, taking medications that would impact tDCS effects (i.e., glutamatergic or GABAergic medications, sodium channel blockers), left-hand dominant, hearing or vision deficits impacting training or tasks, exceeding 80% score on the cognitive training POSIT assessment at the screening visit, and/or chronic medical conditions (i.e., cancer, severe uncontrolled diabetes). The study protocol was approved by the University of Florida Institutional Review Board and written informed consent was obtained from all participants prior to study enrollment following study discussion. All institutional guidelines were followed. Table 1 shows the participant demographics.

**TABLE 1:** Baseline participant characteristics.

	tDCS + CT	Sham + CT	Total	*p*-value
Sample size	12	11	23	−
Age (SD)	72.3 (7.4)	74.2 (7.4)	73.5 (7.2)	0.555
Sex (M:F)	5:7	4:7	9:14	−
Education (SD)	17.1 (2.6)	17.3 (2.9)	17.2 (2.6)	0.869
MoCA scores (SD)	28.2 (1.5)	26.2 (1.7)	27.2 (1.9)	0.008

*tDCS, transcranial direct current stimulation; CT, cognitive training; MoCA, Montreal Cognitive Assessment.*

### Magnetic Resonance Imaging Acquisition

Magnetic resonance imaging data were acquired on a 3-Tesla Siemens Magnetom Prisma scanner with a 64-channel receive array head coil at baseline and 2 weeks. High resolution T1-weighted magnetization-prepared rapid gradient-echo (MPRAGE) structural images were acquired for accurate placement of the MRS voxel and tissue segmentation. Acquisition parameters were repetition time (TR) = 1800 ms, echo time (TE) = 2.26 ms, flip angle = 8 degrees, field of view (FOV) = 240 × 240 × 170 mm, 1.0 mm^3^ isotropic voxel, and scan duration = 183 s.

### Magnetic Resonance Spectroscopy Acquisition

Both GABA and Glx data were acquired in the same MRI session using a MEGA-PRESS sequence from a 3 × 3 × 3 cm^3^ voxel positioned medially on the axial plane aligned with the corpus callosum and superior to the genu of the corpus callosum. Voxel placement was performed by experienced MRS operators, referencing the T1 image. MRS was obtained using the following parameters TR = 2000 ms, TE = 68 ms, flip angle = 90 degrees, FOV = 30 × 40 × 27 mm, 133 pairs of averages, ON editing pulse at 1.9 ppm, OFF editing at 4.68 ppm.

### Magnetic Resonance Spectroscopy Data Analysis

Both Glx and GABA quantification were achieved using GANNET 3.1 in MATLAB (Edden et al., 2014). Siemens.rda files were processed using the RobustSpecReg correction protocol. Briefly, Gannet applies frequency and phase correction to achieve optimal quantification of metabolites to reference values. During processing, for line broadening is achieved *via* exponential apodization, and fast Fourier transform of time-domain acquired data is applied to frequency-domain spectra. Finally, subtraction is used to generate the edited difference spectrum, and extraction of off spectrum. Each metabolite (Glx, GABA) was calculated as its ratio relative of Creatine (Cr), which is set to 3.02 ppm. Cr is a common reference standard in ^1^H-MRS (Jansen et al., 2006) and has been shown to be superior to H_2_O in terms of reliability (Bogner et al., 2010). To correct for tissue-related factors, each volume was controlled for cerebrospinal fluid (CSF) content within the voxel of measurement. This approach is common and has been applied in populations where voxel tissue composition may vary (Harris et al., 2015; Porges et al., 2017).


C=C(1/1-v)CSF0

where *C* is the corrected metabolite concentration, *C*_0_ is the LCModel output, and *v*_*CSF*_ is the volume fraction of CSF contained within the single-voxel spectroscopy (SVS) voxel. This method only considers the partial volume effect of CSF.

**FIGURE 1:**
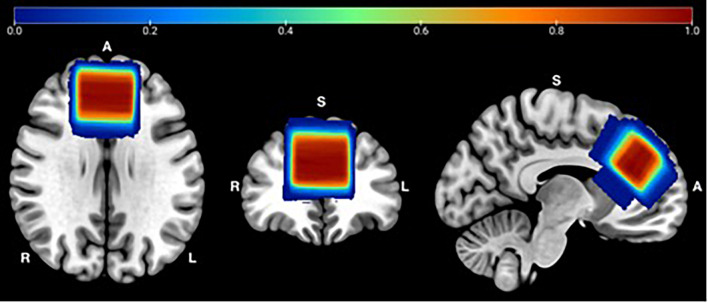
MRS Voxel Overlap: Referencing the T1 image, a single voxel was positioned medially on the axial plane, aligned with the corpus callosum and superior to the genu of the corpus callosum. Abbreviations: R = Right; L = Left; A = Anterior; S = Superior.

Each of the Gannet outputs were visually checked to ensure accurate model fit and voxel placement. A voxel heat map was created to demonstrate MRS voxel overlap between participants across visits, for quality assurance (Figure 1). To achieve this, individual T1 images were segmented by tissue type and warped into Montreal Neurologic Institute (MNI) space using the SPM12 software using the “normalize” procedure with default parameters. The resulting deformation fields were used to warp MRS voxel mask images to MNI space. The normalized maps were combined to one image in MATLAB and displayed using MRIcron (v 1.0.2018) for Mac. Figure 1 demonstrates the accuracy and reliability of MRS voxel placement. Over 99% overlap occurs within the medial frontal lobe for all participants, across the two sessions.

### Transcranial Direct Current Stimulation Parameters and Application

Transcranial direct current stimulation was delivered at 2.0 mA *via* a conventional 1 × 1 tDCS device (Soterix Medical, tDCS-CT for clinical trials). Set-up procedures were identical for both active and sham conditions. Two 5 × 7 cm^2^ saline-soaked Soterix sponge electrodes (0.9% NaCl; 10 ml total/sponge) were placed over the frontal cortices at F3 (cathode) and F4 (anode), using individualized head measurements and International 10–20 system for electrode locations. Prior behavioral findings support the specified montage and intensity, demonstrating the ability to elicit a 2.0 mA net increase and excitability under both the anode and cathode electrodes (Nissim et al., 2019). For each training session, 40-min of computerized cognitive training were applied with active or sham stimulation delivered during the first 20-min. The active group received 20 min of stimulation with 30 s ramp up and down, while the sham group received 30 s of stimulation with 30 s ramp up and down. The 30 s stimulation duration in the sham condition produced the sensation of active simulation without the full duration of stimulation. No significant differences were observed in sensation ratings and blinding efficacy for the sample (previously reported in Nissim et al., 2019).

### Cognitive Training Procedure

The computerized cognitive training program was implemented through POSIT Science BrainHQ, as previously detailed (Nissim et al., 2019). All participants received cognitive training. Participants were randomly assigned to train on four out of eight adaptive tasks in each session, resulting in 40 min of cognitive training per day (10 min per task). All participants received equal number of trainings across the eight tasks. Specifically, the program was composed of four working memory related tasks (Card shark-visual N-Back task, Auditory aces- auditory N-Back task, Memory grid, and To-do-list) and four speed of processing tasks (Double decision- useful field of view, Divided attention, Hawk eye, and Target tracker). Significant cognitive and functional improvements in older adults have been previously validated (Berry et al., 2010) utilizing this program.

### Statistical Analysis

All statistical analyses were performed using SPSS Statistics 25 (IBM, Armonk, NY, United States). Demographic data and neurotransmitter concentration of the two groups (active, sham) were compared at baseline. Separate factorial analysis of covariance (ANCOVAs) were calculated for CSF-corrected GABA:Cr and Glx:Cr concentrations as dependent variables, groups (active, sham) as fixed-factor, and age, MoCA scores, number of intervention days, and baseline GABA:Cr and Glx:Cr concentrations as covariates. Given the hypothesized additive effect of repeated tDCS sessions in metabolite concentrations, the total number of intervention days completed were applied as a covariate. Further, age, baseline neurotransmitter concentrations, and MoCA scores (Reis et al., 2009; de Aguiar et al., 2020) were included as covariates to mitigate their impact on post-intervention concentrations. Mounting evidence within the tDCS and cerebral metabolite literature collectively (Reis et al., 2009; Filmer et al., 2019; de Aguiar et al., 2020) address the importance of accounting for these baseline values.

## Results

### Evaluation for Differences in Baseline Characteristics Between Groups

Twenty-three healthy, older adults completed all data collection visits of the study. Participants that completed more than 80% of the intervention sessions were included in this study. No adverse events were reported during this investigation. Age, sex, and education were not statistically different at baseline between the active and sham groups (Table 1). Descriptive statistics showed a marked difference in MoCA scores between groups at baseline. Therefore, the MoCA score was added as a covariate in the statistical model.

### Effects of Intervention on Glutamate/Glutamine:Creatine Concentrations

**FIGURE 2:**
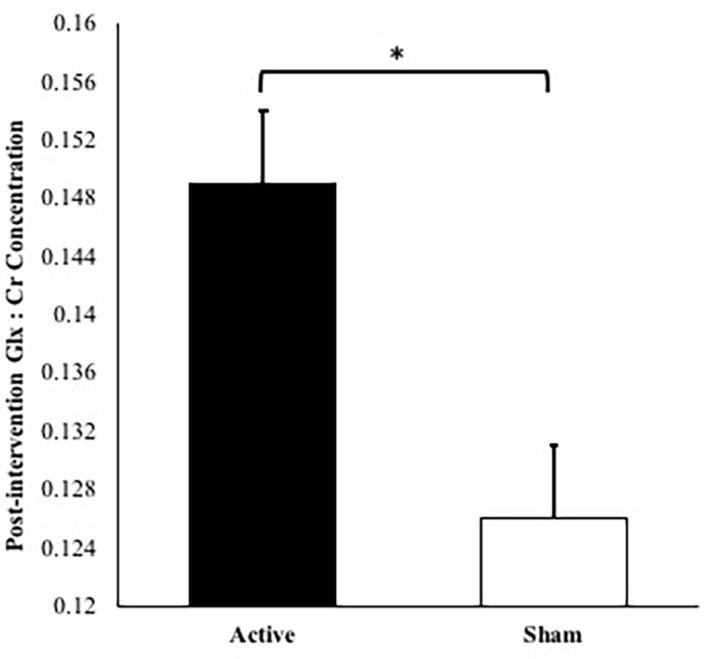
Post-intervention Glx:Cr concentrations for active condition vs. sham condition accounting for age, number of intervention days, MoCA scores, and baseline Glx:Cr concentration (^∗^*p* = 0.010). *Note*: Error bars depict standard errors. Covariates appearing in the model are evaluated at the following values: baseline Glx:Cr = 0.134, age = 73.2, MoCA score = 27.2, number of intervention days = 9.87.

No significant differences were observed at baseline in Glx:Cr concentrations between the active and sham groups, *t*(21) = 2.06, *p* = 0.052. The covariates, MoCA scores and baseline Glx:Cr values, were significantly related to post-intervention Glx:Cr concentrations [*F*(1,17) = 5.61, *p* = 0.030, partial η^2^ = 0.248 and *F*(1,17) = 5.56, *p* = 0.031, partial η^2^ = 0.246, respectively]. There was also a significant effect of intervention group [*F*(1,17) = 8.47, *p* = 0.010, partial η^2^ = 0.332, observed power = 0.783] on post-intervention Glx:Cr values after controlling for age, MoCA scores, number of intervention days, and baseline Glx:Cr concentrations (Figure 2). The overall model including intervention group with age, MoCA scores, number of intervention days, and baseline Glx:Cr concentrations as covariates explained 47.3% of the variance in post-intervention Glx:Cr concentrations.

### Effects of Intervention on Gamma-Aminobutyric Acid:Creatine Concentrations

**FIGURE 3:**
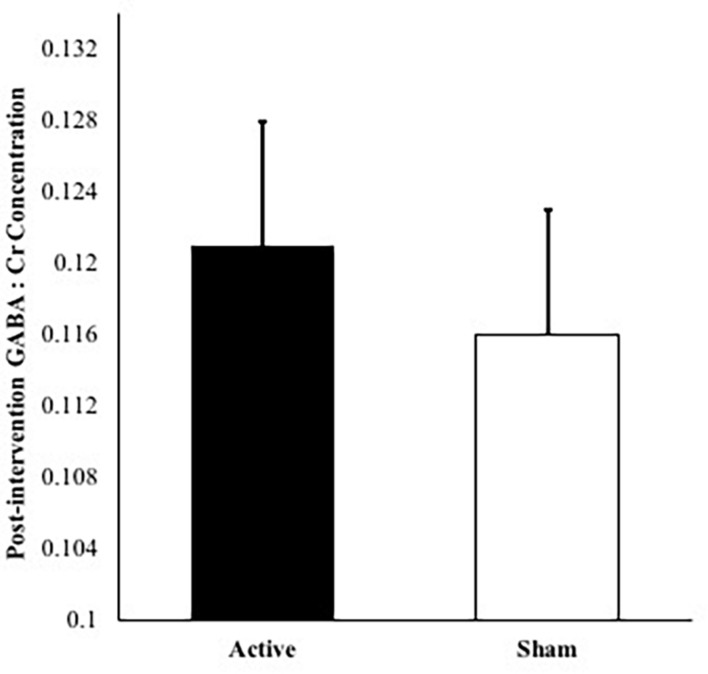
Post-intervention GABA:Cr concentrations for active condition vs. sham condition accounting for age, number of intervention days, MoCA scores, and baseline GABA:Cr concentration. *Note*: Error bars depict standard errors. Covariates appearing in the model are evaluated at the following values: baseline GABA:Cr = 0.117, age = 73.2, MoCA score = 27.2, number of intervention days = 9.87.

No significant differences were observed in GABA:Cr concentrations between active and sham groups at baseline, *t*(21) = 0.491, *p* = 0.629. The covariate of baseline GABA:Cr concentration was significantly related to post-intervention GABA:Cr concentration [*F*(1,17) = 5.95, *p* = 0.026, partial η^2^ = 0.259]. No significant GABA:Cr metabolite difference was evident between active and sham groups post-intervention (*p* = 0.650), after controlling for age, MoCA scores, number of intervention days, and baseline GABA:Cr concentrations. Figure 3 depicts GABA:Cr concentrations values in the active vs. sham groups following 2 weeks. The overall model including intervention group with age, MoCA scores, number of intervention days, and baseline GABA:Cr concentrations as covariates explained 46% of the variance in post-intervention GABA:Cr concentrations.

## Discussion

The current study explored the combined impact of multiple sessions of tDCS and cognitive training on excitatory and inhibitory neurotransmitter concentrations. Frontal lobe Glx:Cr concentrations increased following 2-weeks of paired cognitive training and tDCS intervention over the prefrontal cortex. Meanwhile, the GABA:Cr concentrations in the frontal lobe was similar for each intervention group. Thus, the combined intervention suggest increased excitatory response with tDCS exposure, but no apparent inhibitory response.

To our knowledge, this is the first study assessing Glx and GABA concentrations after a multi-session combined prefrontal tDCS and cognitive training intervention in healthy older adults. Following 2-weeks of active tDCS and cognitive training, Glx:Cr concentrations were significantly increased when controlling for age, MoCA scores, number of intervention days, and baseline Glx:Cr values. The shift toward increased excitatory neurotransmitter concentration in the current study coincides with suggestions that tDCS facilitates changes in glutamate (Cohen Kadosh et al., 2015). Ultimately, this may augment learning when targeting regions involved in skill execution. Namely, the adult brain shifts to facilitate plasticity when introduced with a new stimulus (e.g., tDCS and cognitive training). The excitation during initial skill acquisition allows for the activity-dependent refinements of cortical circuits (Carcea and Froemke, 2013), a contribution to neuronal communication and signal processing that determines learning and memory formation. As such, these changes in excitatory neurotransmitter concentration may further support the notion that longer duration and more persistent adjustments in excitability involve the action of NMDA receptors (Nitsche et al., 2003; Tremblay et al., 2016). Nevertheless, since ^1^H-MRS is not sensitive to variation in NMDA receptors but rather local tissue neurotransmitter concentration (i.e., bound and unbound neurotransmitters), our interpretation is limited.

Alternately, the stochastic resonance hypothesis may provide additional insight into the mechanistic interpretation for improved Glx:Cr concentration. Small amounts of noise added to non-linear systems can increase the quality of the stimulus through stochastic resonance. Stochastic resonance refers to a phenomenon where an optimal level of noise is added to a subthreshold signal, causing the signal to cross the threshold and enhance performance (Moss et al., 2004). van der Groen et al. (2018) found that adding transcranial random noise stimulation (tRNS) bilaterally to visual cortex enhanced decision-making when stimuli were just below perceptual threshold. Hence, tDCS may have the capacity of increasing stochastic resonance within the prefrontal cortex, possibly inducing greater excitability.

Further, understanding what the increases in Glx:Cr concentration might indicate for cognitive performance in older adults complements our results, although not directly within the scope of the current study. Nissim et al. (2019) demonstrated that 2 weeks of active-tDCS paired with cognitive training versus cognitive training alone significantly improved 2-back target accuracy, a commonly used working memory paradigm. These results aligned with prior behavioral findings supporting active-tDCS induced enhancements in N-back working memory performance (Zaehle et al., 2011). Despite an overlapping sample, the authors cannot conclude whether the working memory improvements in the Nissim et al. (2019) study coincide with the measurable biochemical changes within the frontal lobe in the current study. Our study is limited by a reduced sample set due to ^1^H-MRS data processing challenges. Nevertheless, future studies and analyses should assess whether enhancement in Glx:Cr concentrations are related to working memory performance, particularly in a larger sample of healthy, older adults.

Glx and GABA concentrations were quantified using a voxel positioned within the prefrontal cortex, which underlies both working memory and speed of processing abilities. Age-related structural declines in the prefrontal cortex have been associated with worse performance on working memory and speed of processing tasks (Kraft et al., 2020; Evangelista et al., 2021). Further, ^1^H-MRS studies have demonstrated age-related declines in prefrontal GABA and glutamate concentrations, beginning in middle age (Grachev et al., 2001). Increased prefrontal concentrations of Glx may therefore facilitate improvements in working memory and speed of processing performance following combined tDCS and cognitive training targeting these cognitive domains.

Meanwhile, the current study found no significant change in GABA:Cr concentrations following the 2-week intervention. Acute decreases in inhibitory neurotransmitter concentration in the sensorimotor cortex have been observed following single-session M1 stimulation (Stagg et al., 2009, 2011; Kim et al., 2014; Bachtiar et al., 2015; Patel et al., 2019). The current study applies multi-session tDCS to the prefrontal cortex. This distinction is important since reductions in GABA may be specific to certain brain regions during initial stages of adaptation as well as facilitated tasks. Animal research reported a marked and regionally specific reduction of GABA_*B*_ receptor proteins in the prefrontal cortex with age (McQuail et al., 2012). This age-related loss of normal inhibitory function, possibly mediated by GABAergic mechanisms, is likely to produce an atypical response to adaptation and delay the observed changes in GABA. Hence, given the functional specifity of the prefrontal cortex and despite controlling for baseline GABA:Cr concentrations in the current study, 2-weeks may not be the appropriate time range to detect intervention-related changes in prefrontal GABA concentrations.

Although no significant changes in GABA:Cr concentration were detected, this does not conclusively rule out GABAergic changes in response to a combined tDCS and cognitive training intervention. The expected increases in cortical excitability following tDCS have been shown to be multifactorial and driven by modulation of both GABAergic and glutamatergic signaling (Stagg and Nitsche, 2011). Since pharmacological studies indicate that changes induced by tDCS are particularly dependent on GABA receptors (Nitsche et al., 2004), it is possible that subtle changes in GABA are taking place during the 2-week period that we are unable to detect. While this cannot be confirmed in our investigation, there is literature associating reductions in GABA concentration following motor tDCS with faster short-term learning (Stagg et al., 2011). This finding is in line with the hypothesis that long term potentiation-like plasticity within the cerebral cortex is critically dependent on GABA modulation. However, GABAergic inhibition may be particularly necessary to refine already acquired skills rather than forming new ones in an older population.

Of note, other neuromodulating systems are susceptible to glutamatergic processes. For instance, dopamine, serotonin, and acetylcholine have been reported to mediate the effects of tDCS (Kuo et al., 2007, 2008; Nitsche et al., 2009; McLaren et al., 2018). tDCS may positively or negatively regulate these levels by modulating the dopamine system, enhancing acetylcholine transmissions, and suppressing serotonin (Yamada and Sumiyoshi, 2021). These influences are essential for arousal, attention, and cognitive processes, all vital components in the combined intervention implemented in the current study (Kuo et al., 2007). Together, these effects could further alter the balance between excitatory and inhibitory inputs in the brain (Okun and Lampl, 2008), warranting further consideration in subsequent studies assessing the excitatory/inhibitory effects of tDCS and cognitive training.

### Strengths and Limitations

The current study employed a triple blinded randomized control trial studying tDCS and cognitive training. While the current study is limited by a small sample size and lack of a cross-over design, findings presented here may provide important insight for exploration in the context of future larger studies. Despite reduced statistical power for finding other differences or relationships, preliminary findings may provide a greater understanding into neurotransmitter patterns following tDCS interventions.

This study examined the frontal lobe, a brain region disproportionally affected by aging processes. Thus, the results are limited to the cortical region, selected stimulation parameters, and the underlying intrinsic state of the stimulated brain networks. Further, MRS voxel placement variability between each session may exist despite consistent specialist placement and voxel overlap assessment (Figure 1). Finally, ^1^H-MRS, particularly MEGA-PRESS, is an emerging methodology for the study of complex neurotransmitter concentrations. Software for MEGA-PRESS acquisition and data analyses continue to be developed and refined (Bogner et al., 2010; Mullins et al., 2014; Harris et al., 2015). Therefore, differences in MRS analytic approaches may potentially impact Glx and GABA estimates in ways that are not yet fully understood. Future studies of neurotransmitter concentration response to tDCS will help further our understanding of neural mechanisms of change in non-invasive brain stimulation techniques.

## Conclusion

Collectively, this study provides preliminary evidence suggesting that combining cognitive training and tDCS over the prefrontal cortex elicits sustained cortical excitability (after-effects) following 2-weeks of intervention. These effects may be attributable to stimulation of glutamatergic transmission, which may ultimately facilitate learning in task-associated regions. This maintenance in excitatory concentration following 2-weeks of combined intervention is particularly valuable since recent work has questioned the overall usefulness of combining tDCS and cognitive training in older adults for causing improvement in cognitive performance that outlasts the stimulation session itself (Nilsson et al., 2017). The current study provides a novel examination of the neural effects of combining frontal tDCS and cognitive training, potentially contributing to the identification of optimal parameters to enhance future clinical outcomes (Albizu et al., 2020). Our findings support the combination of tDCS and cognitive training as a potential method for altering neurotransmitter concentrations in the frontal cortices, which may have implications for neuroplasticity.

## Data Availability Statement

The datasets presented in this article are not readily available because the data analyzed in this study is subject to the following licenses/restrictions: Deidentified data presented in the manuscript are available upon request. Requests to access the datasets should be directed to the study PI, AW, ajwoods@ufl.edu.

## Ethics Statement

The studies involving human participants were reviewed and approved by University of Florida Institutional Review Board. The parent trial was preregistered in clinicaltrials.gov under NCT02137122. The patients/participants provided their written informed consent to participate in this study.

## Author Contributions

SA-A was responsible for data analysis, data interpretation, and manuscript preparation. EB was responsible for data analysis, data interpretation, and drafting of the manuscript. JK and NN were responsible for data acquisition, data analysis, and conducting intervention. AO’S and AA were responsible for data acquisition and data analysis. AI was responsible for data acquisition and conducting intervention. NE was responsible for critical revision of the manuscript. RC was responsible for study concept and design. EP was responsible for study concept and data interpretation. AW was responsible for study concept and design, critical revision of the manuscript, and study supervision. All authors approved the final manuscript.

## Conflict of Interest

The authors declare that the research was conducted in the absence of any commercial or financial relationships that could be construed as a potential conflict of interest.

## Publisher’s Note

All claims expressed in this article are solely those of the authors and do not necessarily represent those of their affiliated organizations, or those of the publisher, the editors and the reviewers. Any product that may be evaluated in this article, or claim that may be made by its manufacturer, is not guaranteed or endorsed by the publisher.
